# Design of Waveguide Bars for Transmitting a Pure Shear Horizontal Wave to Monitor High Temperature Components

**DOI:** 10.3390/ma10091027

**Published:** 2017-09-04

**Authors:** Jiuhong Jia, Qiyue Wang, Zuoyu Liao, Yun Tu, Shan-Tung Tu

**Affiliations:** Key Laboratory of Pressure Systems and Safety, Ministry of Education, East China University of Science and Technology, Shanghai 200237, China; jhjia@ecust.edu.cn (J.J.); qiyue_wang@outlook.com (Q.W.); Y45150073@mail.ecust.edu.cn (Z.L.); sttu@ecust.edu.cn (S.-T.T.)

**Keywords:** high temperature, waveguide bar, design, non-dispersion, structural health monitoring, ultrasonic guided waves, piezoelectric transducers

## Abstract

Guided wave technique could be a possible method for monitoring components working in high temperature above 350 °C. However, this would require the design of an appropriate waveguide bar to transmit the wave, so that its sensing part is not influenced by the high temperature. In the present study, the shape of waveguide bars is designed based on the analysis of wave source characteristics. The critical frequency-width and frequency-thickness products of waveguide bars are analyzed theoretically and numerically to transmit the zeroth shear horizontal wave SH0* in bars. The results show that waveguide bars can cut off all the other wave modes when their frequency-thickness products are smaller than the critical value fd*, and frequency-width products are not smaller than the critical value fw*. Six waveguide bars are designed and fabricated based on the design criteria, and an experiment system is set up to check their work performance. The testing results indicate that the wave signals of the SH0* mode propagate clearly in waveguide bars, and cut off all the other modes when the frequency-thickness products and frequency-width products of the bars meet the design criteria. It is also demonstrated that the dependency of the experimental group velocity of each waveguide bar on frequency is in good agreement with the numerical result. High-temperature experiments also validate the reliability of the designed waveguide bars. Therefore, the critical frequency-thickness product and frequency-width product can be the basis for the design of the waveguide bars.

## 1. Introduction

High temperature components are applied comprehensively in aerospace and process industries. Many researchers have suggested that monitoring the structural health of components with permanently installed transducers is a useful way to maintain their safety [1,2,3,4,5,6,7,8,9]. Permanent installation will allow measurements to be more frequent and remove errors introduced by re-installing transducers. The financial consequences of prolonged operational shutdowns have previously aroused strong interest in developing ultrasonic transducers that can operate at elevated temperatures above 350 °C for a long time. Li [10] has developed BiScO3-PbTiO3 (BS-PT) high-temperature transducers. However, lithium niobate decreases in sensitivity over time when left in high temperature. Hou et al. [11] have directly deposited thick piezo films onto high temperature structures. Sinding et al. [12] have sprayed piezoelectric powder on the surfaces of structures. The attachment procedure for these transducers is intricate and time-consuming, requiring several deposition steps and subsequent poling to achieve robust attachment. None of them have achieved ideal results for high temperature longtime usage. 

Therefore, some non-contact technology has also been developed, such as laser-ultrasonic wave technology and electromagnetic acoustic transducers. Applications of laser-ultrasonic wave technology are covered in different research areas, and include defect inspection [13] and online monitoring [14]. However, its applicability in the industrial field is limited because of the comparatively expensive price and characteristics that are easily influenced by external disturbance. Electromagnetic acoustic transducers do not require couplant for transmitting sound, which makes them suitable for the inspection of very hot and very cold parts [15,16,17]. However, whether a electromagnetic acoustic transducer is based on the Lorenta force [18] or the magnetostrictive principle [19], most of these transducers require strong magnets, and so the transducer tends to be bulky and heavy, which make it not suitable for structural health monitoring (SHM) [20]. 

To overcome the above-mentioned deficiencies, the most promising inexpensive method is to use a buffer waveguide bar to isolate the fragile transducers from the hot or corrosion specimens. Such an implementation enables the application of commercially available standard piezo-crystals as transducers. As a result, many researchers have studied the design of the waveguide bars. Lawrence [21] has announced a system that uses thin wire to minimize dispersion, but the transmitted power propagating into the specimen is too low. In order to overcome some of the problems of a single thin wire, Lawrence [22] has changed to a bundle of thin wires. Nevertheless, cross-talk between individual wires may complicate the signal analysis, and there are practical difficulties associated with attaching each individual wire to the test structure. Jen [23] has disclosed a tapered ultrasonic waveguide bar with an external layer of attenuation cladding. The cladding can remove the effects of the waveguide bar’s boundaries by damping and limiting surface reflections. However, the signal is slightly delayed, distorted, and strongly attenuated. Heijnsdijk [24] has disclosed a coiled foil waveguide bar. The thickness of the foil is arranged to be much smaller than the smallest wavelength of the propagated signal, thus satisfying the low frequency-dimension product for non-dispersive transmission. This coiled foil is better suited to extensional rather than torsional waves. Peter [25] has disclosed an elongate strip of ultrasound transmissive material, which transmitted some shear modes of ultrasonic waves with notable non-dispersion, but it isn’t suitable for longitudinal wave. Kwon [26] has designed a tapering waveguide bar. When the tapered waveguide bar is adopted, the surviving lower shear horizontal wave can carry most of the transmitted power into the specimen. Due to the squared bottom of the waveguide bar, it seems more suitable for a flat structure rather than a cylindrical structure. According to these research studies, it is understood that the geometric structure of the waveguide bar is the key parameter affecting the dispersion and scattering of waves. However, the quantitative relation between the geometry of waveguide bars and the dispersion of waves has not been investigated. Consequently, the aim of the present study is to provide a criterion for the structural design of the waveguide bars.

## 2. Selection of a Guided Wave Source

Part of the core technology of applying waveguide bars to monitor high temperature components is that the signal transmission should be as strong as possible, and with as little distortion as possible. When the transmitted power into components is strong enough, the signals can transmit from the transducers to the tested component and back again. When the ultrasonic signals travel in the waveguide bars without distortion, the algorithm to evaluate damage to components is relatively simple and easy. In order to reach the core technology, the transmitting energy and directivity of the excited wave are critical to the selection of the wave source. 

The torsional point loading and anti-plane shear loading can only excite shear waves [7]. All the energy of loading is concentrated on the shear waves, and the signal strength of the wave is strong. As a result, the torsional point loading and anti-plane shear sources exhibit the desirable characteristics to be put into use in the waveguide monitoring systems. Moreover, the source radiating equally in all directions is the ideal condition for the guided wave monitoring system. The directivity of the torsional loading is formed into two hemispherical lobes, whereas the anti-plane shear line source radiates equally and strongly in all directions. That is to say, the anti-plane shear sources exhibited the more desirable characteristics than the torsional point sources for the guided wave monitoring system. 

## 3. Structural Design of Waveguide Bars

### 3.1. Theoretical Analyses

**Figure 1:**
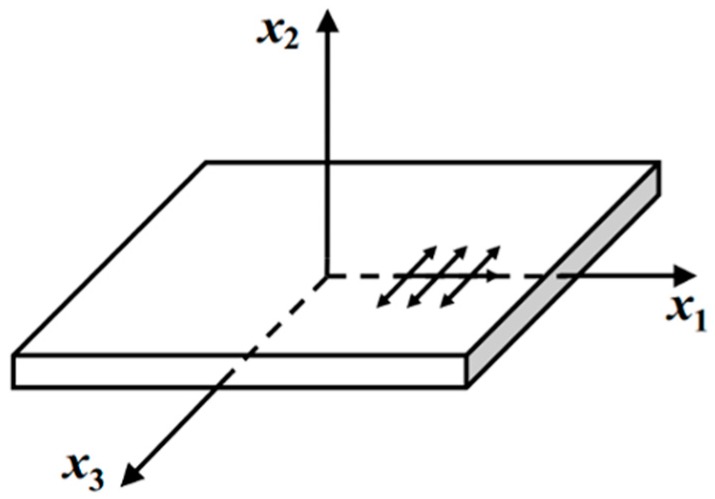
Shear horizontal (SH) wave mode propagation.

The anti-plane shear line loading source doesn’t exist in real life. A waveguide bar of large width-to-thickness ratio (width >> thickness) is the closest practically implementable approximation. The wave excited by the anti-plane shear line loading source is a shear horizontal wave (shorten for SH wave), which can be depicted in Figure 1. The wave propagates in the *x_1_* direction, the particle vibrates in the *x_3_* direction, and there is no out-of-plane particle displacement in the vibration of the SH wave.

The explicit solutions for the group velocity in terms of frequency-thickness product *fd* are constructed by Rose [27]. The group velocity *C_g_* is

(1) $$C_{g}\left(fd\right)=C_{T}\sqrt{1-\frac{{\left(n/2\right)}^{2}}{{\left(fd/C_{T}\right)}^{2}}}\;\left[fd\geq {\left(fd\right)}_{n}\right],$$

where *d* equals to the thickness of the layer, *f* is frequency, *C_T_* is the shear wave speed. *n* ∈ {0, 1, 2, ⋯}.

**Figure 2:**
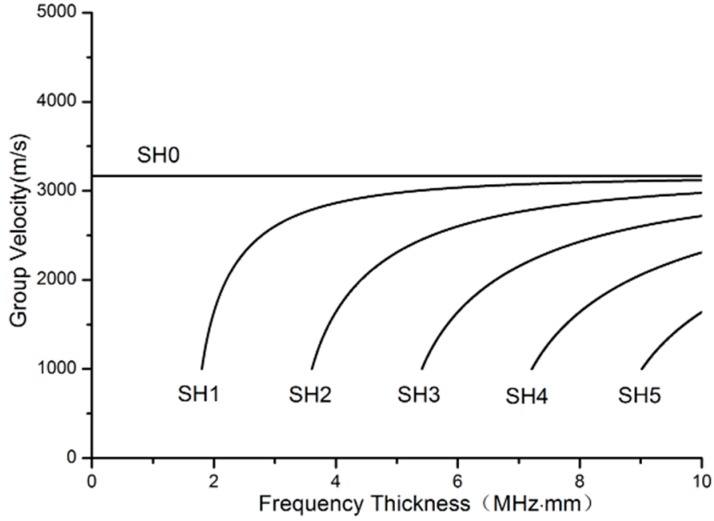
SH mode group velocity dispersion curves for the stainless steel layer.

Since the material of many high temperature components is made of stainless steel, stainless steel is also chosen as the material of waveguide bars in order to reduce refraction of the wave transmitting from waveguide bars into components. The shear wave speed in a stainless steel layer is *C_T_* = 3200 m/s. The group velocity curves for the first six SH modes in a stainless steel layer are plotted in Figure 2. 

### 3.2. Calculating the Thickness

According to Figure 2, when *n* = 0, the wave is in the SH0 mode, the group velocity of which is not frequency-dependent. It is a non-dispersion wave propagating at the shear wave speed *C_T_*. All other SH modes (*n* ∈ {1, 2, ⋯}) are dispersive. As the frequency-thickness product *fd* approaches infinity for any given fixed n, the group velocity of any SH mode approaches that of bulk shear waves *C_T_*. In this case, it is different to achieve in actual engineering. Here, we consider that the frequency-thickness product is small enough. 

Supposing that the group velocity of SH1 is zero, Equation (1) can be written as

(2) $$C_{g}\left(fd\right)=C_{T}\sqrt{1-\frac{\left(1/2\right)}{{\left(fd/C_{T}\right)}^{2}}}=0,$$

In this case, a cut off frequency-thickness product can be calculated according to Equation (2)

fd* = 1.6 MHz·mm, (3)

When the frequency-thickness product *fd* is smaller than 1.6 MHz·mm, there is only SH0. Since there is no out-of-plane particle displacement in a SH wave, it is less affected by the presence of surrounding media. Furthermore, the group velocity of the SH0 wave mode isn’t frequency-dependent, which can simplify the signal analysis for different acquisition frequencies. The characteristic that the SH0 wave will not convert to other modes when defects exit can reduce the complexity of data processing and improve the ability to identify defects. Therefore, the SH0 mode is preferred in structural health monitoring over all the other SH modes. The SH0 mode is defined as a desired mode, and all the other SH modes are defined as undesired ones in the present study. The frequency-thickness product fd* is designated as the critical value of the frequency-thickness product to cut off the undesired modes. 

**Figure 3:**
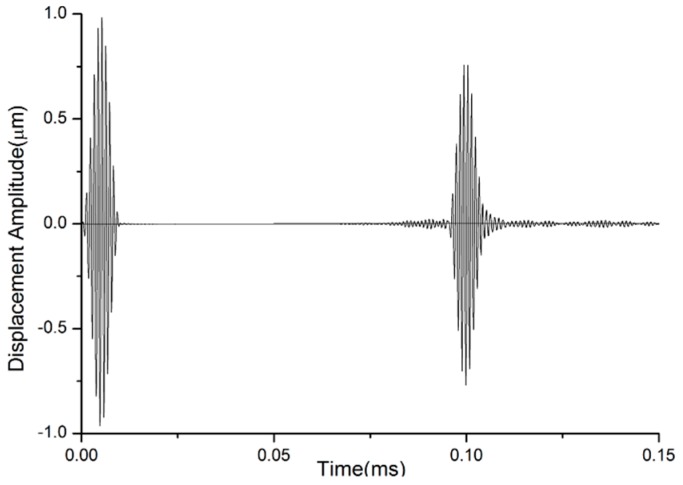
Simulated waveforms in a waveguide bar (thickness 1 mm and width 15 mm).

Useful frequencies for non-destructive inspection normally range from 1 MHz to 5 MHz [25]. When the acquisition frequency of non-destructive monitoring is selected, the thickness of the layer can be calculated according to the critical value of the frequency-thickness product. For example, when the signal frequency is 1 MHz, the thickness *d* can be calculated as 1.6 mm, according to Equation (3). A geometrical thickness of the layer equal to 1 mm can be selected, taking into account the convenience of material selection. At this condition, the undesired SH modes can be cut off and only SH0 can propagate through the layer with 1 mm thickness, as shown in Figure 3. The first wave packet is excitation signal, and the second is received signal. The received signal is very clear. The presence of other modes that are much weaker than the main signal can be ignored. 

Therefore, the critical frequency-thickness product can be considered as the criterion for thickness selection, when the signal frequency is selected. 

### 3.3. Calculating the Width

**Figure 4:**
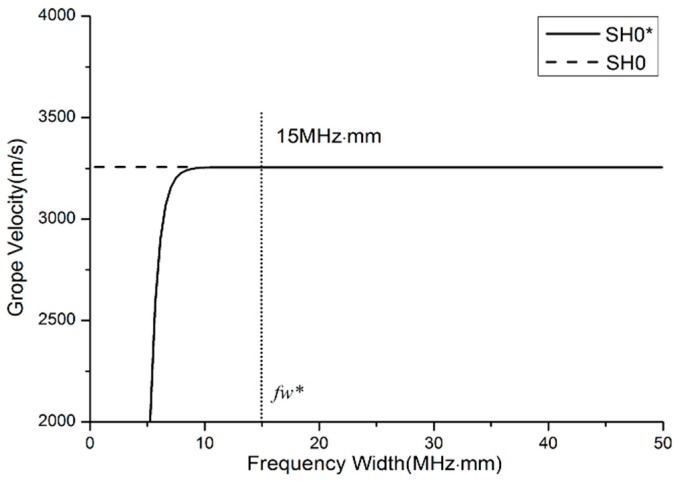
Simulated group velocity dispersion curves against the frequency-width product.

Dispersion characteristics in flat layers and in waveguide bars are not identical. Strictly speaking, the term ‘shear horizontal’ doesn’t make sense in a waveguide bar other than an infinite plate. Hence, the name SH0* mode is chosen to indicate the zeroth-order SH mode in a waveguide bar. In order to compare the SH0 and SH0* modes, the group velocities of the waveguide bar with 1 mm thickness are simulated by ANSYS/LS-DYNA software (ANSYS 12.0, ANSYS, INC., Canonsburg, PA, USA). The material of the waveguide bar is 316 L steel. The material’s characteristics are listed in Table 1. Three waveguide bars are designed, and the geometrical sizes to be analyzed by numerical simulation are inventoried in Table 2. In the process of simulation, excitation signals are sent on one end, and reception signals are caught on the same end of bars. The reception signals are part of excitation signals that travel along the waveguide bar and conduct back from the end of the waveguide bars. The group velocities of waveforms are calculated by the method of Time of Flight [28]. The group velocities’ curves versus the frequency-width product of the reception signals are calculated and shown in Figure 4. It is found that the dispersion behavior in stainless steel waveguide bars is a function of the product of the frequency of the signal and the width of the waveguide bars. All SH0* curves of waveguide bars with different widths are coincident. At frequency, well above the cut-off fw* = 15 MHz·mm, the group velocity of SH0* modes asymptotically approaches the bulk shear velocity of the SH0 mode. The waves also propagate clearly with advantageous non-dispersion, which can be noticed from Figure 3 in Section 3.2.

Therefore, the critical frequency-width product can be the criterion for width design to acquire the ideal wave mode.

### 3.4. Frequency Dependence

**Figure 5:**
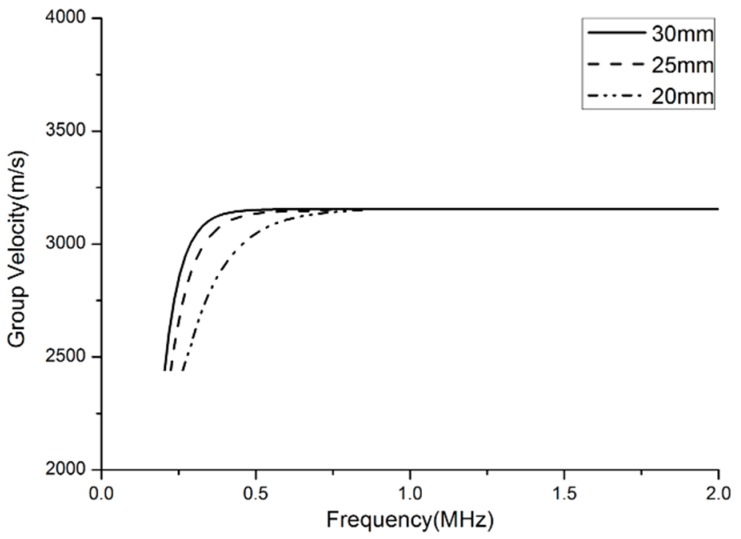
Group velocity dispersion curves against frequency.

The curves of the group velocity dispersion versus frequency for the SH mode of 1-mm thick steel bars of different widths (30, 25 and 20 mm) are plotted in Figure 5. It is found that the group velocity dispersion has a cut-off frequency that depends on the width of the bars. At frequencies well above the cut-off, the group velocity asymptotically approaches the bulk shear velocity *C_T_*. Based on the dispersion characteristics of the SH wave in waveguide bars, it can be found that the signal frequency and the geometric width of waveguide bars are strongly interrelated at low frequency-width products. Therefore, the signal frequencies in the application of non-destructive testing should be high enough to make sure that the frequency-width product isn’t lower than the critical value. In this condition, the waves propagating through the waveguide bars can be guaranteed to be in the SH0* mode. 

### 3.5. Structural Design Criteria

Based on the above-mentioned analysis, the critical frequency-thickness product and frequency-width product can be the design criteria of the geometrical structure of the waveguide bar for a given frequency in the normally used non-destructive frequency range (1 MHz, 5 MHz). When the frequency-thickness products are smaller than the critical value fd*, and frequency-width products are not smaller than the critical value fw*, waveguide bars can cut off the undesired wave modes. For the waveguide bars designed by these criteria, the SH0* mode of ultrasonic waves can propagate through with advantageous non-dispersion. It is an ideal condition to detect the deterioration process of components. 

## 4. Room Temperature Experimental Verification

### 4.1. Experimental System

**Figure 6:**
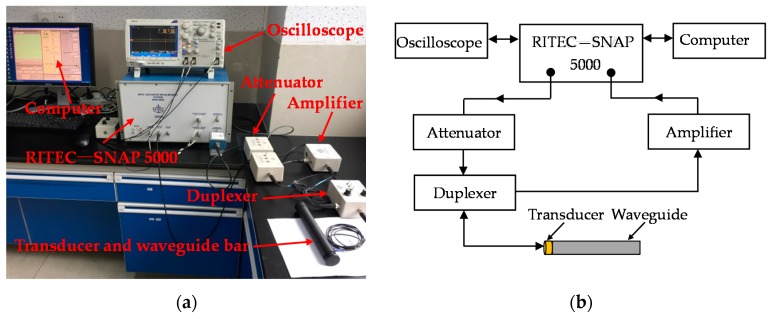
Room temperature experimental system: (**a**) Experimental set-up; (**b**) System sketch map.

**Figure 7:**
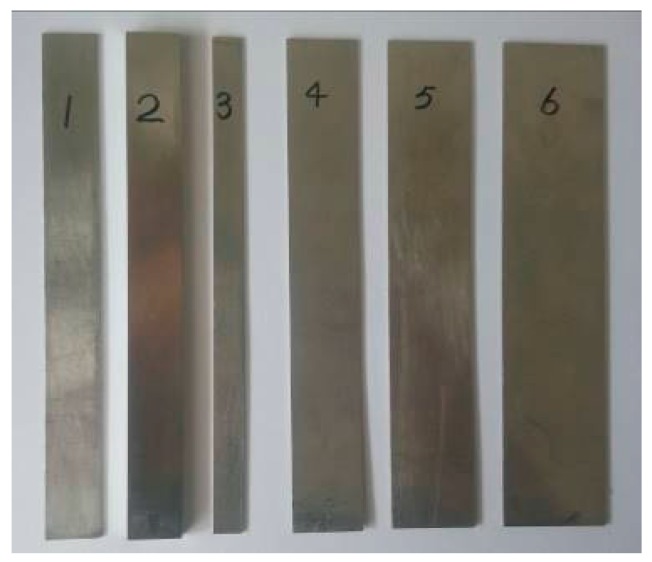
Waveguide bars.

**Figure 8:**
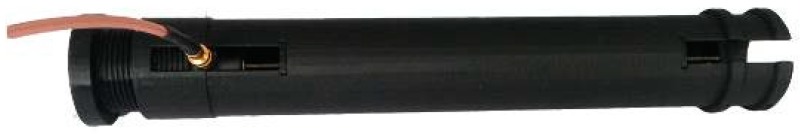
Installation of the waveguide bar and transducer.

In order to verify the reliability of the design criteria regarding the frequency-thickness product and the frequency-width product, and thus design the geometrical structure of the waveguide bar, an experimental system is set up by consulting references [29,30]. The diagram and the picture are shown in Figure 6. The experimental system includes an ultrasonic testing system RITEC-SNAP 5000 (Ritec, INC., Warwick, RI, USA), an oscilloscope, an attenuator, an amplifier, a duplexer, a waveguide bar, and a transducer. The transducer is the Olympus V153-RM (Olympus NDT, INC., Waltham, MA, USA), which has a central frequency of 1 MHz and can excite shear horizontal waves. The duplexer is the RDX-6 (Ritec, INC., Warwick, RI, USA), which is a transformer arrangement that delivers high-power pulses to a transducer, while returned signals from the same transducer are transferred to a receiver. It can also filter signals at the same time. Ten cycle tone bursts modulated with a Hanning window are generated at 1 MHz using the testing system, and recorded at a sampling rate of 50 MHz. Moreover, six different waveguide bars are fabricated, and the pictures are shown in Figure 7. The geometric structures of the bars are listed in Table 3. A purpose-made installation tool is designed to fix the transducer on one end of the waveguide bar. In the process of testing, the transducer works as exciter and receiver. The installation picture of transducers is evinced in Figure 8. The SWC shear wave couplant is applied between the transducer and the end of the waveguide bar in experiments.

### 4.2. Verification of Thickness

**Figure 9:**
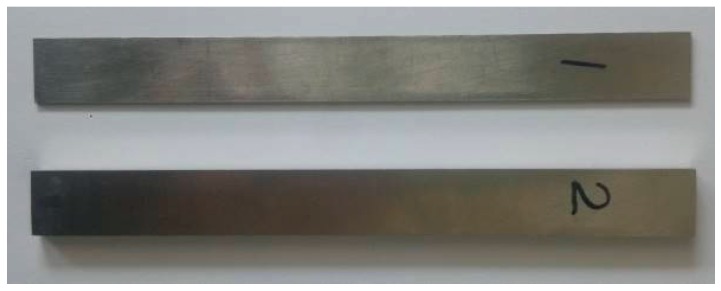
Waveguide bars named No. 1 and No. 2 (the thickness of No. 1 is 1 mm, and the thickness of No. 2 is 4 mm).

**Figure 10:**
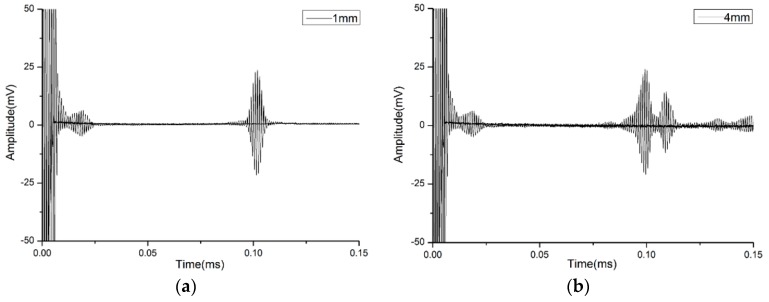
The reception signals of No. 1 and No. 2 waveguide bars: (**a**) Thickness 1 mm; (**b**) Thickness 4 mm.

We laid special stress on analyzing the reception signal at one of the normally used frequencies, which is 1 MHz. In order to prove the effect of the thickness of waveguide bars on the purity of the signal, two waveguide bars have been designed and fabricated. The width of the waveguide bars is designed as 15 mm, according to the critical frequency-width product fw*. The thicknesses of the bars are chosen as 1 mm and 4 mm, respectively. When the thickness is 1 mm, the frequency-thickness product *fd* = 1 MHz·mm, which is smaller than the critical frequency-thickness product fd*. When the thickness is 4 mm, the frequency-thickness product *fd* = 4 MHz·mm, which is bigger than the critical frequency-thickness product fd*. The picture of the waveguide bars is shown in Figure 9. The experimental waveforms of waveguide bar 1 and waveguide bar 2 are plotted in Figure 10. There is cross-talk at the beginning of the time domain of the waveforms. In the present study, the cross-talk signals are not analyzed; instead, the reception signals back from the ends of the waveguide bars are analyzed. In Figure 10a, there is a main reception signal, and the presence of other modes is much weaker than the main signal, so that the reception signal can be considered as only one mode, and all other modes are cut off. The calculated group velocity 3012 m/s is in good agreement with the theoretical group velocity 3200 m/s of the SH0 wave in the steel layer, so the received signal is in SH0* mode. It is noticed that for the received signals in Figure 10b, waveforms are dispersive. The group velocity of the first one packet is calculated as 3016 m/s, and the group velocity of the second one is calculated as 2740 m/s, which is in good agreement with the theoretical group velocity 2752 m/s of SH1 mode. There are still other modes following after SH1 wave mode. 

In other words, when the real frequency-thickness product of the waveguide bar is smaller than the critical value, only SH0* can propagate through. Otherwise, the reception signal will disperse. Therefore, the critical frequency-thickness product fd* = 1.6 MHz·mm can be the design criterion for a given signal frequency.

### 4.3. Verification of Width

**Figure 11:**
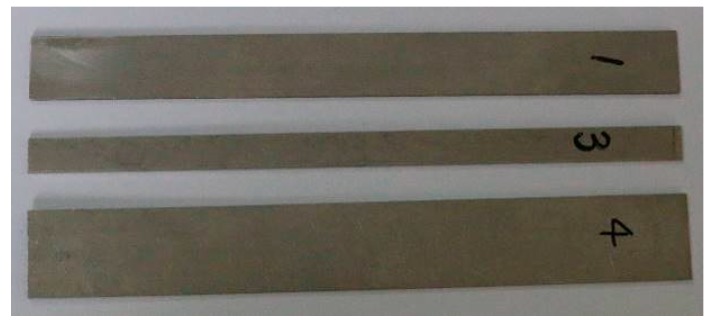
Waveguide bars named by No. 1, No. 3 and No. 4 (the width of No. 1 is 15 mm, No. 3 is 7 mm, and No. 4 is 20 mm).

**Figure 12:**
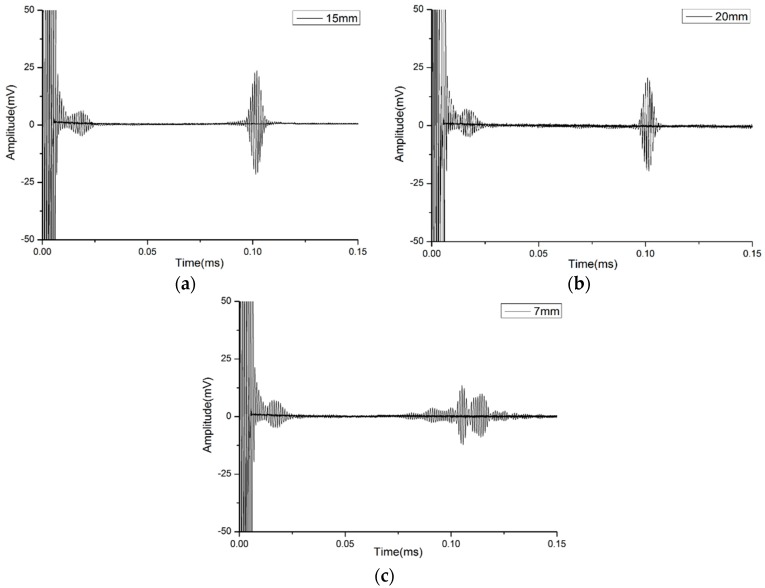
The reception signals of No. 1, No. 3 and No. 4 waveguide bars: (**a**) Width 15 mm; (**b**) Width 20 mm; (**c**) Width 7 mm.

In order to prove the effect of the width of waveguide bars on the purity of the reception signal, two waveguide bars have been designed and fabricated. The thickness of the bars is designed as 1 mm, which is based on the critical frequency-thickness product fd*. The width of the waveguide bars is 7 mm and 15 mm, respectively. When the width is 7 mm, the frequency-width product *fw* = 7 MHz·mm, which is smaller than the critical frequency-width product fw*. When the width is 15 mm, the frequency-width product *fw* = 15 MHz·mm, which is equal to the critical frequency-width product fw*. The picture of the waveguide bars is shown in Figure 11. The signals propagated through waveguide bar No. 1, No. 3 and No. 4 are plotted in Figure 12. In Figure 12a,b, only the SH0* mode exists, and all of the other SH modes are cut off. In Figure 12c, waveforms are dispersive, and more than one SH mode exists in the reception signals.

Therefore, when the real frequency-width product of the waveguide bar isn’t smaller than the critical value, only the SH0* mode can propagate through. Otherwise, the reception signal will disperse. Therefore, the critical frequency-width product fw* = 15 MHz·mm can be the design criterion for a given signal frequency.

### 4.4. Verification of the Frequency Dependence

According to the critical frequency-width product fw* = 15 MHz·mm, three steel waveguide bars are chosen. They are No. 4, No. 5 and No. 6. For the signal frequency 1 MHz, the frequency-width products are all bigger than the critical value fw*.

**Figure 13:**
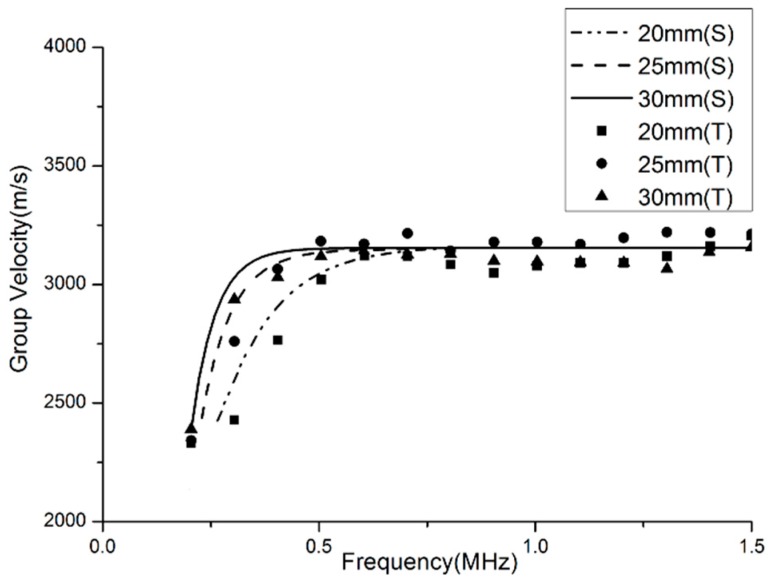
Comparison of group velocity dispersion curves between simulation and test; Notes: S—simulation data; T—testing data.

The group velocities of the different wave modes excited in all those three waveguide bars are calculated by time of flight method at different frequencies. The experimental results and numerical results are compared in Figure 13. The experimental data proximately distribute around their corresponding numerical values; that is to say, they are in good agreement with each other. 

According to Figure 13, it is also found that the wave signals propagating in the rectangular waveguide bar have a cut-off frequency that depends on the width of the waveguide bars. For the bars with different widths, they have a different cut-off frequency. For a given frequency, the designed width needs to be wider than the calculated width, so that the real frequency-width product isn’t lower than the critical value, even when the signal frequency fluctuates in engineering application.

### 4.5. Propagating Characteristics of Designed Waveguide Bars

**Figure 14:**
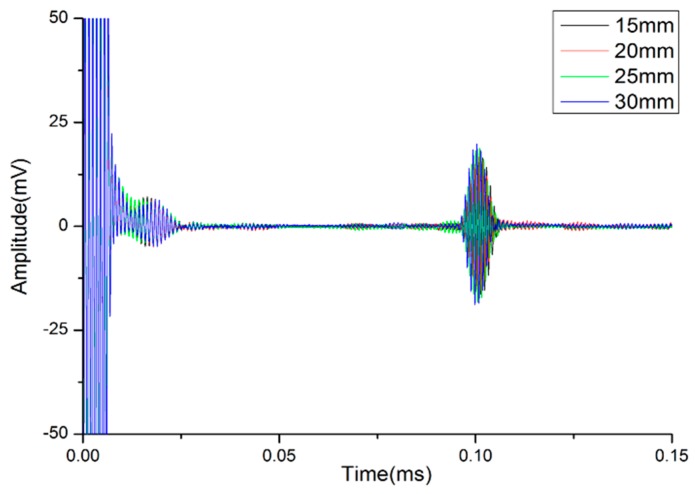
The reception signals of waveguide bars (No. 3–No. 6).

The frequency-thickness products and frequency-width products of the waveguide bars (No. 3–No. 6) all meet the design standards. The experiments are performed, and reception signals are plotted in Figure 14. Waveforms are very clear with significant non-dispersion. The presence of other modes that are much weaker than the SH0* mode can be ignored. 

Therefore, it has been verified that the critical frequency-thickness product fd* and frequency-width product fw* can be the basis to design the geometrical structures of waveguide bars in order to get pure SH0* mode signal. 

## 5. High Temperature Experimental Validation

**Figure 15:**
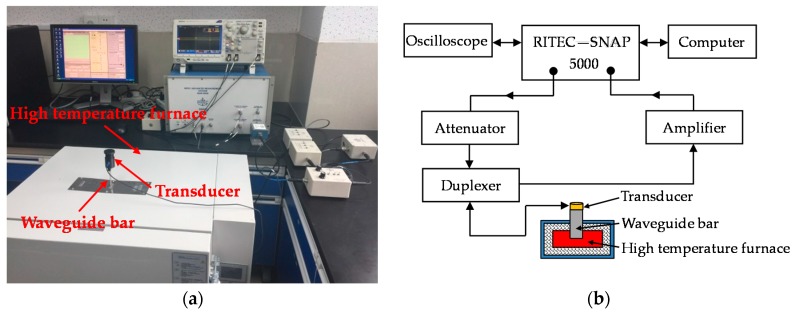
High temperature experimental system: (**a**) Experimental set-up; (**b**) System sketch map.

Propagating characteristics of designed waveguide bars are then tested at high temperature. In this testing system, a high temperature furnace is applied. The test set-up is shown in Figure 15. The waveguide bar goes through a hole in the insulating layer of the furnace and reaches outside of the furnace. One end of the waveguide bar is bonded inside the furnace, and the other end is coupled to the ultrasonic transducer, which works in room temperature. The waveguide bars designed in the present study can be integrated in ultrasonic testing equipment to monitor high-temperature components. The range temperature of this ultrasonic testing equipment mainly depends on the material of the waveguide bar. The material selected for waveguide bars is 316 L steel, which can be used up to 650 °C. Therefore, the ultrasonic testing equipment is expected to operate up to 650 °C.

**Figure 16:**
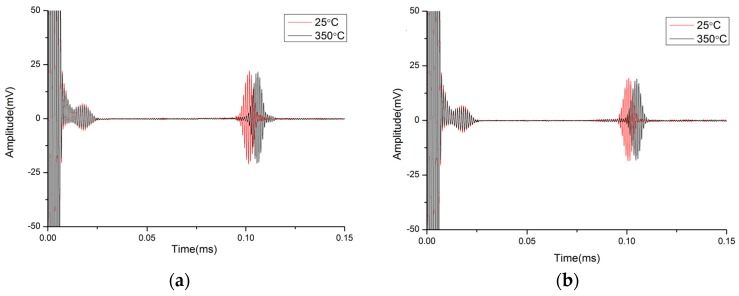
The received signals of waveguide bars: (**a**) Waveforms of No. 1; (**b**) Waveforms of No. 4.

In the present study, the target temperature of 350 °C is tested. When the furnace is heated to 350 °C, the temperature is holding. The transducer end can be safely held by hand. There is no noticeable increase in temperature. Due to the limitation of high-temperature installation tools, the propagating characteristics of two waveguide bars have been tested, which are No. 1 and No. 4. The received signals are compared with ones from room temperature experiments, as shown in Figure 16. It is noticed that the received signal is very clear, too. The presence of other modes of signals that are much weaker than the main signal can be ignored. These features are in line with those of the room temperature experiment. Signal amplitudes remain strong. There is no drastic change in attenuation. However, differences still exist. The waveforms delay about 4% at higher temperature because of the reduction of group velocity in the waveguide bar at high temperatures.

Generally, the temperature of high-temperature components fluctuates slightly in a normal working period, so the change of group velocity can be negligible. When the temperature fluctuates obviously, the temperature compensation technology can be utilized. Therefore, the conclusion can be drawn that the waveguide bars designed by the critical frequency-thickness product fd* and frequency-width product fw* can be used to measure high-temperature components by pure SH0* mode signal. 

## 6. Conclusions

In order to introduce ultrasonic wave technology into high-temperature components monitoring to improve their security, the waveguide bars have been designed to transmit the wave so that the sensing part is not influenced by high temperature. According to wave source characteristics analysis, a large aspect ratio rectangular waveguide bar is designed to approximately load the anti-plane shear line source. In order to get a very clear wave signal with advantageous non-dispersion in the waveguide bar, the transmitting characteristics are analyzed theoretically and numerically. It is noticed that the frequency-thickness product of bars should be smaller than the critical value fd*, and frequency-width product should be not smaller than the critical value fw* to cut off the undesired wave mode. Moreover, some waveguide bars are designed and fabricated based on these design criteria, and experiments are carried out. The experimental dependencies of group velocities on frequencies are in good agreement with numerical simulation results. It is also found from the experimental waveforms that the signals can propagate clearly and non-dispersedly in the waveguide bar when the frequency-thickness products and frequency-width products of the bars meet the design criteria. High temperature experiments are carried out, and the experimental results show that the designed waveguide bars can work quite well. Therefore, the feasibility of the design method is verified. 

## Figures and Tables

**Table 1:** The material characteristics of 316 L steel.

Elastic Modulus	Poisson’s Ratio	Material Density
E = 211 Gpa	R = 0.286	d = 7800 kg/m^3^

**Table 2:** The geometrical sizes of waveguide bars for simulation.

Number	Width/mm	Thickness/mm	Length/mm
1	20	1	150
2	25	1	150
3	30	1	150

**Table 3:** The geometrical sizes of waveguide bars for test.

Number	Width/mm	Thickness/mm	Length/mm
No. 1	15	1	150
No. 2	15	4	150
No. 3	7	1	150
No. 4	20	1	150
No. 5	25	1	150
No. 6	30	1	150
